# Supplementary figures and images for: Reduced production of the major allergens Bla g 1 and Bla g 2 in Blattella germanica after antibiotic treatment
Source: PLoS One. 2021 Nov 23;16(11):e0257114. doi: 10.1371/journal.pone.0257114 (PMC8610280; doi:10.1371/journal.pone.0257114)

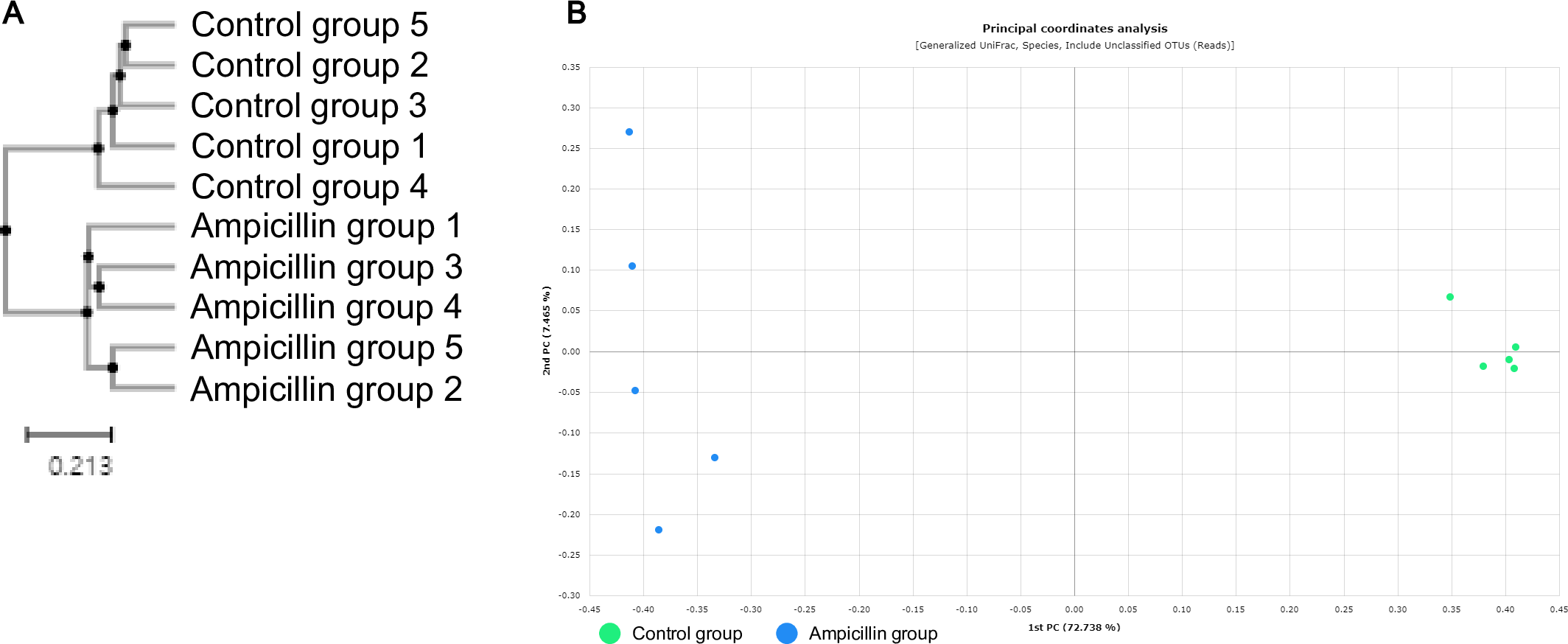

Supplement: S1 Fig — (A) UPGMA (unweighted pair group method with arithmetic mean) clustering. (B) Principal coordinates analysis depicting differences in the taxonomic compositions of the bacterial communities among the two groups. (TIF) [file pone.0257114.s001.tif]

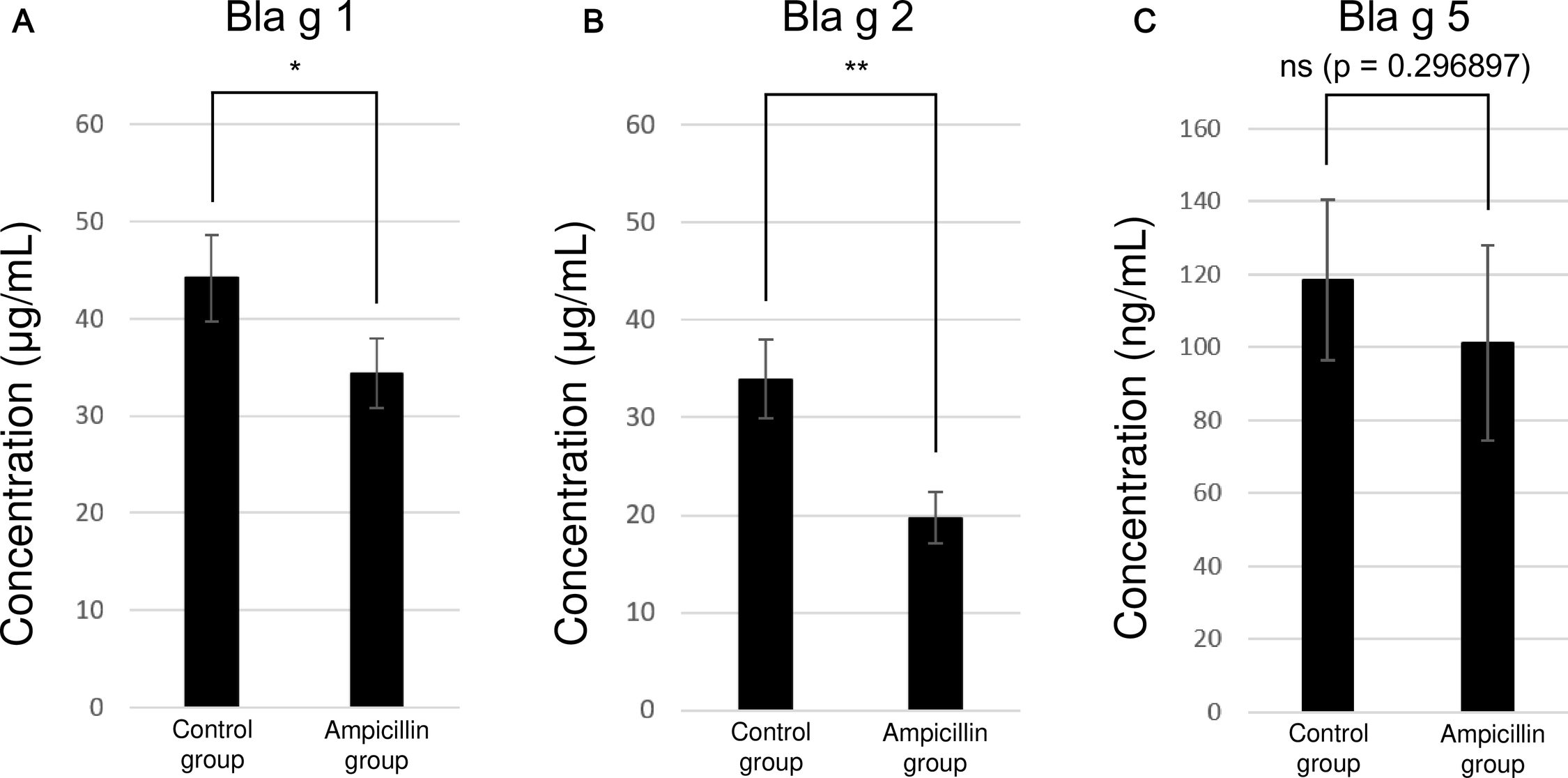

Supplement: S2 Fig — Concentrations of (A) Bla g 1, (B) Bla g 2, and (C) Bla g 5 in the extracts were measured using enzyme-linked immunosorbent assays. (TIF) [file pone.0257114.s002.tif]
