# Supplementary material for: Reduced production of the major allergens Bla g 1 and Bla g 2 in Blattella germanica after antibiotic treatment
Source: PLoS One. 2021 Nov 23;16(11):e0257114. doi: 10.1371/journal.pone.0257114 (PMC8610280; doi:10.1371/journal.pone.0257114)
Supplement: Attachment — Submitted filename: Reviewer Recommendation and Comments for Manuscript Number PONE.docx [file pone.0257114.s004.docx]

**Reviewer Recommendation and Comments for Manuscript Number PONE-D-21-26586**

The authors analysed and compared between the microbiomes of the German cockroach (*Blattella germanica*) treated with ampicillin and the untreated control. The aim for the antibiotic treatment was to reduce the total bacterial population that may directly or indirectly be involved in allergen production found in the cockroach. This would assist in producing protein (allergen) extracts with minimal presence of the bacteria for immunotherapy.

Overall, the manuscript was well-written. The microbiome data analyses based on 16S rRNA amplicon sequencing and RNA-seq were thorough. An adequate number of samples and statistics were also included. However, whilst the manuscript is technically sound, there are several issues that I would like to highlight to the authors. Addressing these comments by the authors would hopefully help to strengthen their manuscript. The comments are categorised under major, minor and miscellaneous as follows:

Major comments

1. In the introduction, the authors stressed that resident bacteria play a role in the insect’s growth and reproduction, and antibiotic treatments alter its microbiome composition. The authors then presented their results that supported the latter. They showed that antibiotics brought about several changes to the bacterial population, microbiome composition, gene expression and allergen (bla protein) quantification, etc. These are all justified.

However, the underlying motivation of this study (i.e. through antibiotic treatment of *B. germanica*), as stated by the authors (in the abstract, introduction and discussion), was to obtain the allergen protein extracts with “minimal amount of bacteria” for immunotherapy purposes. The authors brought this argument only towards the end of the introduction (lines 81-88), and mentioned this aim very briefly in the discussion (lines 320-321) without providing a strong justification for the antibiotic treatment. I wondered if filter sterilising the crude extracts (line 146) would solve the problem i.e. to obtain the protein extracts without bacteria and without needing the antibiotic application. If filter-sterilising the extracts do not work, why not?

In addition, the justification/rationale for the microbiome analyses were weak and were not even highlighted at the end of the introduction despite these analyses being the major results of this study. What questions were the authors attempting to answer by performing the microbiome analyses? What was the rationale?

The authors need to address the above gaps by providing and elaborating on the reasoning behind the study and analyses presented. The justification should be clarified in the introduction and re-stated in the discussion, and should be linked to the results in a meaningful way. Doing so serves as a fundamental basis to any scientific study including this one.

2. The discussion section, although in general was well-written, lacked the depth necessary to understand the results in a more meaningful way. Most of what was written in the discussion is a reiteration of the results. For example, it is clear that the antibiotic treatment gave rise to ‘super-resistant’ taxa (*Desulfovibrio* and *Plantomycetes*) in/on the cockroach, and the control cockroach contained *Blattabacterium* as the main taxon (lines 327-354). However, how does these results relate to the Bla protein production quantified in the study or other insights that might be derived from the results? The authors could extend this discussion by e.g. stating that it is probably *Blattabacterium* that may be involved in the allergen production by the cockroach directly/indirectly and provide evidence based on the other results and those of past studies related to the study’s main objective.

The arguments presented for the RNA-seq results (lines 355-368) were also equally weak. The authors found that there was a “significant difference in the molecular functions related to catalytic activity and binding” (line 366) but did not link these to anything relevant in this study. Gene expression of the microbiome was found higher in antibiotic-treated cockroach compared to control, but can the authors speculate why this was so?

The authors discussed that ampicillin may have influenced the production of allergens directly or indirectly (lines 390-392), which I agree, based on their results. However, would it be possible to do some correlation analyses between the results the authors obtained to support this statement?

The authors went on by stating that a limitation of the study was that *Blattabacterium* is not yet culturable. However, there are methods that the authors can use e.g. qPCR, FISH, to study the bacterium’s behaviour against the bacterial population. These can be explored and mentioned.

Minor comments:

1. Some of the results, e.g. Figs. 5 and 9, only re-iterated the message of the earlier figure(s) and thus could be better presented as supplementary data. This ensures that the section is more concise.
2. In addition to the results, there are also data that the authors should provide as supplementary. These were mentioned as ‘data not shown’ (lines 99, 296). Having these data available are in line with PLOS ONE’s principles on data transparency, availability and reproducibility.
3. Some of the methods need to be a bit more elaborated, particularly on ELISA (lines 150-151) and TRIZOL RNA extraction (Line 154).
4. Some sub-sections in the methods need to be condensed, re-organised and have its format readjusted. Repetition was found for sample processing (Line 107-112), RNA extraction/cDNA libraries (Lines 153-157; Lines 178-184), statistical analyses (Lines 130-141; lines >200). Reorganisation/merging the sub-sections would make it less repetitive and ensure that readers can follow the procedures better.

Miscellaneous:

Line 62: Reference to the author is missing

Line 82: What do the authors mean by “suitable” protein extract? What is considered “suitable”? This comment links to the first major comment of this review.

Line 85-86: The effect of cockroach on what? The same also goes for line 395: ‘the effects of *Blattabacterium”* on what? And line 402: To investigate the effect of bacteria on patients in what sense? These need more clarification.

Line 99: Add ‘the’ before ‘concentration’

Line 117-118: Format adjustment required

Line 127: Table caption should be placed before the table

Line 130-131: “Bioinformatic analyses”, not “bioinformatics analyses”

Line 279-280: The authors mentioned that the 1236 DEGs doubled, but which one doubled? Was it in ampicillin-treated group? Please clarify.

Line 295-296: “The expression of Bla g 2 RNA more than doubled in the ampicillin-treated group”. Isn’t this ironic compared to the results presented in Figs. 8 and 9? What is the difference between this result not shown and the results presented in Fig. 8? Aren’t they both RNA-seq of the *bla* gene?

Line 352: Ratio of bacteria to what?

Line 358-359: What did the authors mean by “clustering occurred first among the control group”?

Line 369-370: This opening statement is confusing when the authors stated “…to confirm changes in the allergens according to the changes”. Please rephrase this.
