# Supplementary material for: Reduced production of the major allergens Bla g 1 and Bla g 2 in Blattella germanica after antibiotic treatment
Source: PLoS One. 2021 Nov 23;16(11):e0257114. doi: 10.1371/journal.pone.0257114 (PMC8610280; doi:10.1371/journal.pone.0257114)
Supplement: Attachment — Submitted filename: Response to Reviewers.docx [file pone.0257114.s006.docx]

**Responses to Reviewer Comments**

For reviewer 1.

1. The rational of using G1 cockroaches

🡪 We have included following sentence:

(Lines 106–107 in MATERIALS AND METHODS) We used G1 cockroaches because we had to administer ampicillin immediately after hatching.

2. The reduction of fertility after ampicillin treatment

🡪 We have included following sentence:

(Lines 233–235 in RESULTS) The number of laid eggs did not vary between the groups, but the offspring were reduced approximately ten times after ampicillin treatment.

3. No morphological differences between the cockroaches

🡪 We have included following sentence:

(Lines 235–236 in RESULTS) In addition, no morphological differences were observed between the groups.

4. The justification to use only sonification for protein extraction

🡪 We have included following sentence.

(Lines 150–151 in MATERIALS AND METHODS) Defatting was not performed as there was little fat in the sample and to minimize bacterial contamination.

5. The future plan to study the effect or ampicillin treatment to the adult cockroaches

🡪 We have included following sentence:

(Lines 428–429 in DISCUSSION) Furthermore, a comparative study on the effect of ampicillin treatment on the microbiome and allergen production between adult and nymph cockroaches is needed.

For reviewer 2.

Further, I am not convince that comment 2 from reviewer#2 (However, how does these results relate to the Bla protein production quantified in the study or other insights that might be derived from the results?) had been properly addressed. Also, has the link between Bla protein and microbiome been reported previously? If yes, please elaborate in discussion.

Finally, please provide reference for the the assertion that "Blattabacterium may play a key role in allergen production in cockroaches".

To the best of our knowledge, the effect of the microbiome on cockroach allergens or that of antibiotic treatment on allergens has not yet been reported. However, in *Riptortus pedestris,* the absence of the endosymbiont, *Burkholderia* spp., led to a decrease in vitellogenin (the reference for this study has been added to the manuscript). Vitellogenin was reported as an allergen in *B. germanica*; however, *Burkholderia* spp. was not detected in our *B. germanica* samples.

Based on our results, we inferred that "*Blattabacterium* may play a key role in allergen production in cockroaches"; therefore, there was no reference provided.

🡪 The following sentences have been added in the manuscript.

(Lines 408–414 in DISCUSSION) In this study, treatment with ampicillin eliminated *Blattabacterium*, an endosymbiont of *B. germanica*, and reduced the production of several allergens. This suggests that *Blattabacterium* may play a key role in allergen production in cockroaches either directly or indirectly. To the best of our knowledge, the effect of the microbiome on cockroach allergens has not been reported. However, in *Riptortus pedestris,* the absence of the endosymbiont *Burkholderia* spp. led to a decrease in vitellogenin, an allergen in *B.* *germanica* (26).
